# Supplementary material for: Spermidine-Eugenol Supplement Preserved Inflammation-Challenged Intestinal Cells by Stimulating Autophagy
Source: Int J Mol Sci. 2023 Feb 18;24(4):4131. doi: 10.3390/ijms24044131 (PMC9964041; doi:10.3390/ijms24044131)
Supplement: Supplementary file 1 [file ijms-24-04131-s001.zip › ijms-2165329-supplementary.pdf]

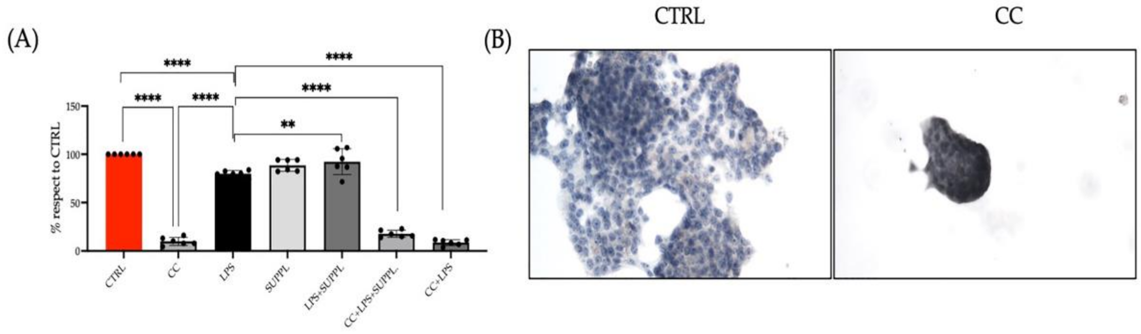

Supplementary Figure S1. (A) Cell viability (MTT) after a 24 h exposure of NCM460 cells to the supplement (SUPPL, 1.2 μM spermidine [SPD] + 92 μM eugenol (EUG), lipopolysaccharide (LPS, 1 ng/mL) and Compound C (CC, 5 μM) either alone or in combination compared to the untreated control (CTRL). (B) Comparison of the control cells and cells treated with CC over 24 h showing very few cells with the remainder disintegrated. CC was administered 30 min prior to the addition of the SUPPL and SUPPL pretreatments were for 1 h prior to LPS exposure for 24 h. The number of stars *, **, *** and **** represents significant differences between treatments as determined by one-way ANOVA at the 99% confidence level ($p < 0.01$). The black dots indicate the positioning of the individual replicates within the bar for each sample.
